# Impact of Vacuum-Assisted Venous Drainage on Forward Flow in Simulated Pediatric Cardiopulmonary Bypass Circuits Utilizing a Centrifugal Arterial Pump Head

**DOI:** 10.21470/1678-9741-2019-0311

**Published:** 2020

**Authors:** Daniel Peres Guimarães, Luiz Fernando Caneo, Gregory Matte, Luciana P. Carletto, Valéria Camargo Policarpo, Ana Vitória C. X. Castro, Matheus H. C. Miranda, Priscila S. Costa, Marcelo B. Jatene, Idagene Cestari, Fabio B. Jatene

**Affiliations:** 1 Cardiovascular Surgery Division, Instituto do Coração do Hospital das Clínicas da Faculdade de Medicina da Universidade de São Paulo (InCor-HC-FMUSP), São Paulo, SP, Brazil.; 2 Department of Cardiac Surgery, Boston Children’s Hospital, Boston, MA, USA.

**Keywords:** Congenital Heart Disease, Cardiopulmonary Bypass, Child, Vacuum, Drainage, Pressure, Erythrocytes

## Abstract

**Objective:**

To analyze the impact of vacuum-assisted venous drainage (VAVD) on arterial pump flow in a simulated pediatric cardiopulmonary bypass circuit utilizing a centrifugal pump (CP) with an external arterial filter.

**Methods:**

The simulation circuit consisted of a Quadrox-I Pediatric oxygenator, a Rotaflow CP (Maquet Cardiopulmonary AG, Rastatt, Germany), and a custom pediatric tubing set primed with Lactated Ringer's solution and packed red blood cells. Venous line pressure, reservoir pressure, and arterial flow were measured with VAVD turned off to record baseline values. Four other conditions were tested with progressively higher vacuum pressures (-20, -40, -60, and -80 mmHg) applied to the baseline cardiotomy pressure. An arterial filter was placed into the circuit and arterial flow was measured with the purge line in both open and closed positions. These trials were repeated at set arterial flow rates of 1500, 2000, and 2500 mL/min.

**Results:**

The use of progressively higher vacuum caused a reduction in effective arterial flow from 1490±0.00 to 590±0.00, from 2020±0.01 to 1220±0.00, and from 2490±0.0 to 1830±0.01 mL/min. Effective forward flow decreased with increased levels of VAVD.

**Conclusion:**

The use of VAVD reduces arterial flow when a CP is used as the main arterial pump. The reduction in the forward arterial flow increases as the vacuum level increases. The loss of forward flow is further reduced when the arterial filter purge line is kept in the recommended open position.

An independent flow probe is essential to monitor pump flow during cardiopulmonary bypass.

**Table:** 

Abbreviations, acronyms & symbols	
CP	= Centrifugal pump
CPB	= Cardiopulmonary bypass
CVR	= Cardiotomy venous reservoir
GME	= Gaseous microemboli
HLMs	= Heart-lung machines
KAVD	= Kinetic-assisted venous drainage
RP	= Roller pump
RPM	= Rotations per minute
VAVD	= Vacuum-assisted venous drainage
VLP	= Venous limb pressure

## INTRODUCTION

Traditional gravity siphon drainage with small size cannulae and tubings may provide insufficient blood return to the cardiotomy reservoir and compromise effective forward flow and tissue perfusion^[1,2]^. Assisted venous drainage techniques such as kinetic-assisted venous drainage (KAVD) and vacuum-assisted venous drainage (VAVD) are used to improve venous return^[3]^. KAVD uses a centrifugal pump (CP) placed in the venous line to generate negative venous line pressure while VAVD involves application of a constant vacuum pressure to the airtight cardiotomy venous reservoir (CVR). VAVD is largely used not only in minimally invasive surgery, but also commonly in pediatric cardiac surgery, with small circuits and/or small cannulae^[3]^.

Roller pump (RP) and CP are the two main arterial pump designs used during cardiopulmonary bypass (CPB). CP employs the mechanical principle of a constrained vortex created by rotating nested cones or vane impellers. This design eliminates circuit tubing compression and mitigates shear stress-induced cellular responses and complements activation, which potentially improves hemostasis^[4,5]^. This lack of occlusion also reduces local areas of negative pressure cavitation and gaseous microemboli (GME) transmission^[6]^. Some authors have concluded that the use of CP may reduce CPB-related morbidity and decrease the cost of cardiac surgery^[7]^. The use of CP rather than RP during regular CPB may be justified based on the theoretical advantages of CPs over RPs^[5-7]^.

Until now, to the best of our knowledge, there is no evidence-based difference in hematologic parameters and clinical outcomes with the use of a CP or RP as the main arterial pump in adult cardiac surgery^[8]^. Despite no superior advantage of CPs over RPs, CPs are used in 51% of CPB cases in Brazil, in both pediatric and adult patients^[9]^. There are centers using CPs in almost 100% of their cases, which is justified only by their observational outcomes. Interestingly, VAVD is routinely used in almost every case, utilizing a CP as the arterial pump head. CP is non-occlusive and could facilitate bubble transgression when used in the arterial position associated with VAVD. Jegger et al.^[10]^ demonstrated that VAVD is a safe technique as long as the perfusionist stops the vacuum when the arterial pump is no longer in use. That group also concluded that the use of RP is preferred in the arterial position^[10]^. Another potential problem associated with the use of CPs and VAVD is the influence of vacuum on the effective forward flow, either due to increased arterial filter purge flow or a reduced CP output with VAVD applied to the CVR. These concerns mean that perfusionists must monitor forward flow with a flow probe distal to any potential circuit shunts.

This study aimed to evaluate the effective forward flow using a CP as the arterial pump associated with VAVD, with and without an external arterial line filter purge in use, in a simulated pediatric CPB circuit.

## METHODS

### Experimental Circuits

**Fig. 1:**
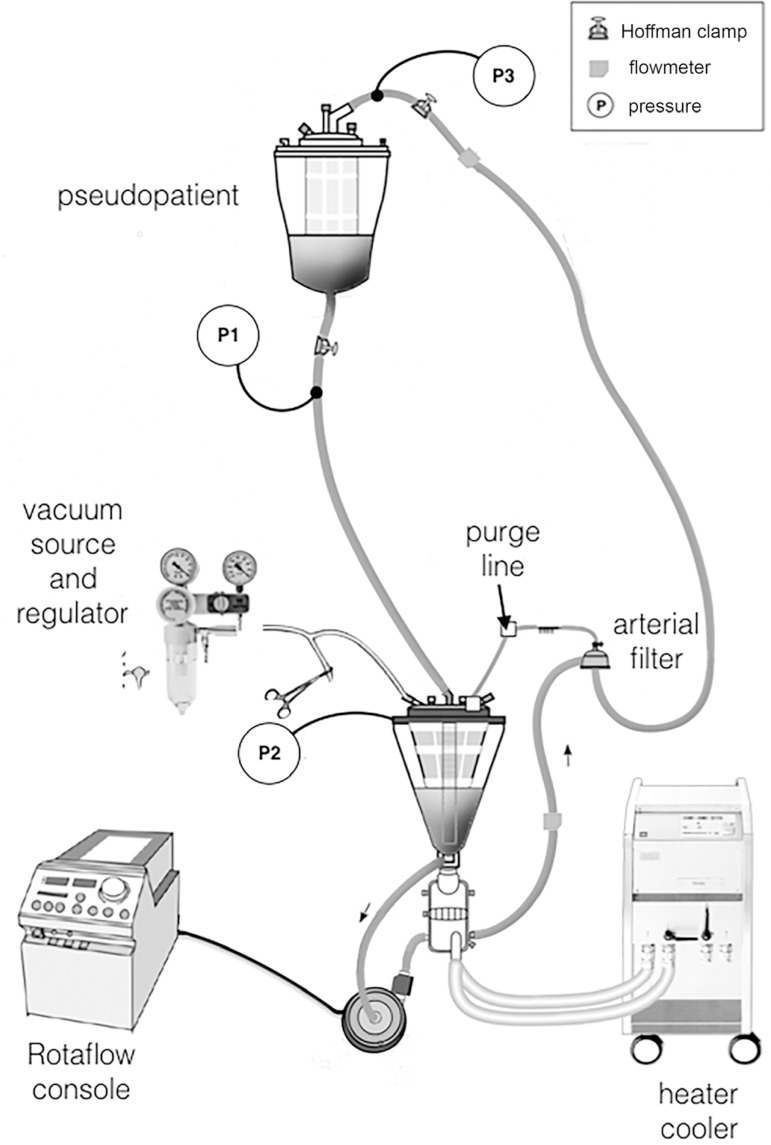
The circuit employed in this study simulating a pediatric cardiopulmonary bypass with a centrifugal pump as the main pump. Two Hoffman clamps were used to control the venous drainage and the post-cannula pressure (adjusted to 50 mmHg at the beginning of each range of flow - 1500, 2000, and 2500mL). Two flow probes were placed into the arterial line before and after the arterial filter. Pressure was measured in three sites: at the venous limb close to the pseudopatient (P1), at the cardiotomy venous reservoir (P2), and post-arterial cannula site (P3).

The experimental circuit included a pediatric Quadrox-iD oxygenator without an integrated arterial filter (Maquet Cardiopulmonary AG, Rastatt, Germany), a Rotaflow CP as the arterial pump, an 18Fr straight tip arterial cannula (Medtronic, Inc, Minneapolis, MN, USA), an HCU-20 heater cooler system (Maquet Cardiopulmonary AG, Rastatt, Germany), a vacuum regulator (Braile Biomedica, Brazil), and a pediatric arterial line filter (Braile Biomedica, Brazil), as shown in Figure 1. This filter was added into the arterial arm of the circuit with an 8 mm inner diameter tubing purge line.

The pseudopatient consisted of a 2000 ml capacity hard-shell reservoir (Maquet Cardiopulmonary AG, Rastatt, Germany). This reservoir level was located 80 cm above the CVR and was connected with 3/8” venous tubing (Figure 1). Venous limb lengths were standardized to 120 cm. The arterial pump consisted of a Rotaflow CP for all test conditions connected to 150 cm of ¼” ID tubing.

### Experimental Design

The experimental circuit was primed with Lactated Ringer's solution (Baxter, São Paulo, Brazil) followed by heparinized human packed red blood cells to achieve a circuit hematocrit of 30%. The pseudopatient’s volume was held constant at 300 mL using a Hoffman clamp just before the venous line. Pseudopatient’s pressure (post-arterial cannula pressure) was maintained at 50 mmHg at the beginning of the study using another Hoffman clamp after the arterial cannula. The oxygenator venous reservoir level was kept at a minimum of 200 mL during all experiments. A vacuum regulator (Nipro Corporation do Brasil, SP, Brazil) controlled VAVD pressures.

Venous limb pressure (VLP), CVR pressure, and arterial flow were recorded in five different conditions: atmospheric pressure (vacuum off) and four different vacuum levels (aiming a negative pressure of -20, -40, -60, and -80 mmHg - conditions A to E), Tables 1 and 2. Initial pump flow rate was adjusted to 1500, 2000, and 2500 mL/min with purge line clamp closed. The experiment was conducted with the purge line both open and closed to compare the deviated flow through the arterial line filter purge line.

**Table 1:** Mean venous limb pressure (P1), mean venous reservoir pressure (P2), and arterial line flow (forward flow) measured at baseline flow of 1500, 2000, and 2500 mL/min with vacuum off and increments of vacuum-assisted venous drainage levels (-20, -40, -60, and -80; conditions A to E) keeping the purge line closed.

Adjusted flow	Condition	P1 venous limb pressure (mmHg)	P2 venous reservoir pressure (mmHg)	Effective forward flow (mL/min)
1500 ml/min	A	-27.67±0.12	-0.73±0.00	1490±0.00
	B	-50.22±0.35	-21.47±0.49	1300±0.00
	C	-67.73±0.11	-39.29±0.08	1100±0.00
	D	-88.01±0.28	-58.70±0.25	880±0.00
	E	-112.25±0.10	-79.72±0.13	590±0.00
2000 ml/min	A	-26.40±1.43	-1.24±1.38	2020±0.01
	B	-51.40±1.92	-24.88±1.59	1810±0.02
	C	-66.08±0.38	-37.79±0.44	1640±0.01
	D	-87.03±0.20	-58.31±0.10	1440±0.00
	E	-109.59±0.19	-80.65±0.10	1220±0.00
2500 ml/min	A	-25.32±0.09	-1.37±0.12	2490±0.00
	B	-41.61±2.42	-19.62±1.39	2370±0.02
	C	-64.32±0.38	-38.40±0.35	2180±0.01
	D	-88.99±0.08	-62.02±0.10	1980±0.00
	E	-107.32±0.01	-80.54±0.09	1830±0.00

**Table 2:** Mean venous limb pressure (P1), mean venous reservoir pressure (P2), and arterial line flow (forward flow) measured at baseline flow of 1500, 2000, and 2500 mL/min with vacuum off and increments of vacuum-assisted venous drainage levels (-20, -40, -60, and -80; conditions A to E) keeping the purge line open.

Adjusted flow	Condition	P1 venous limb pressure (mmHg)	P2 venous reservoir pressure (mmHg)	Arterial flow (mL/min)	Deviated flow (mL/min)
1500 ml/min	A	-29.43±0.11	-0.73±0.00	1230±0.00	430±0.00
	B	-53.15±0.25	-22.52±0.27	960±0.00	490±0.00
	C	-69.17±0.23	-37.80±0.18	740±0.00	550±0.00
	D	-92.47±0.40	-59.48±0.53	440±0.00	610±0.00
	E	-112.28±0.17	-82.04±0.15	160±0.00	650±0.00
2000 ml/min	A	-28.29±0.30	-1.22±0.01	1700±0.01	490±0.01
	B	-46.87±0.11	-18.62±0.34	1480±0.01	560±0.01
	C	-67.49±0.40	-38.63±0.11	1640±0.01	580±0.01
	D	-92.42±0.35	-62.62±0.12	1040±0.00	610±0.00
	E	-111,79±0.17	-81.37±0.13	770±0.01	660±0.01
2500 ml/min	A	-29.43±0.11	-29.43±0.11	1230±0.00	560±0.00
	B	-53.15±0.25	-53.15±0.25	960±0.00	590±0.01
	C	-69.17±0.23	-69.17±0.23	740±0.00	610±0.01
	D	-92.47±0.40	-92.47±0.40	440±0.00	690±0.00
	E	-112.28±0.17	-112.28±0.17	160±0.00	710±0.00

### Data Acquisition

Two Transonic ultrasound flow probes (Transonic Systems, Inc., Ithaca, NY, USA) were used for each set of test conditions in setup I. One flow probe was located before arterial line filter and the other after the filter, as shown in Figure 1.

Three Edwards TruWave disposable pressure transducers (Edwards Lifesciences Corp., Irvine, CA, USA) were placed. The first was located at the beginning of the venous limb (P1), the second at the CVR (P2), and the third at the post-arterial cannula site (P3). Pressure transducers were connected to CPB-100 pressure monitors (Bioengineering Division, InCor-HC-FMUSP, São Paulo, Brazil). Pressure monitor and flowmeter outputs were connected to a DataQ DI-710 data acquisition device (DataQ, Akron, OH, USA) and then connected to a computer via universal serial bus port. WinDaq data acquisitions software (DataQ, Akron, OH, USA) was used to record real-time data at 1000 samples per second per channel. A 30 s segment of pressure and flow waveforms was recorded for each set of variables.

### Purge Line Shunt Flow Calculation

The purge line shunt flow was calculated in setup II with the purge line both open and closed. Purge line shunt flow was calculated from measured flow rates at pre-oxygenator and pre-arterial cannula locations: purge line shunt flow = pre-arterial filter flow - post-arterial filter (pre-arterial cannula) flow.

### Statistical Analysis

The variables are presented in mean and standard deviation. One-way ANOVA-repeated measures were used to compare total pressure drop between venous reservoir levels (1500 mL, 2000 mL, and 2500 mL) and VAVD levels (0, −20, −40, −60, and −80). Tukey’s multiple comparisons test was done to identify the difference between the variables studied. The statistical significance threshold was a *P*-value < 0.05. All analyses were performed using GraphPad Prism software (San Diego, CA, USA) for Mac version 6.0 (Microsoft Corporation, Redmond, WA, USA).

## RESULTS

**Fig. 2:**
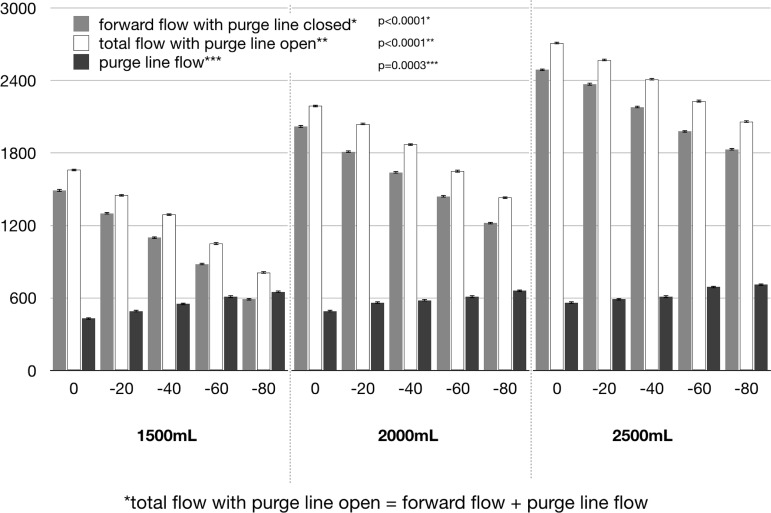
The total (post-arterial filter flow + deviated flow through the purge line) forward flow increases when purge line is open as a compensation to the stealing flow in this situation.

**Fig. 3:**
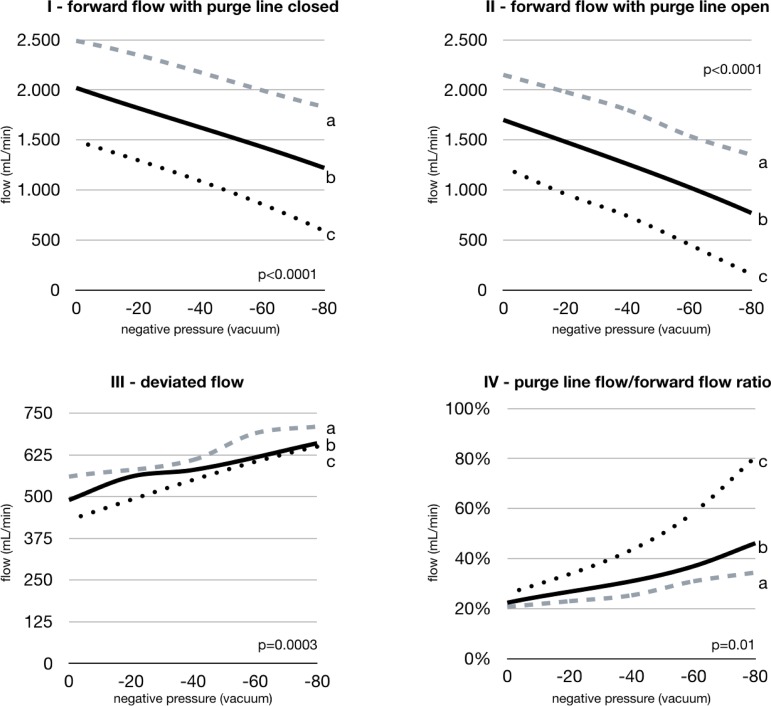
Influence of the negative pressure applied to venous reservoir and to the initial forward flow (arterial line flow) of 2500 mL/min (a) , 2000 mL/min (b), and 1500 mL/min (c); with the purge line closed (I) and purge line open (II); to the deviated flow (III); and percentage of purge line flow/forward flow ratio (IV).

The mean VLP (P1), mean venous reservoir pressure (P2), effective forward flow, and purge line shunt flow are shown in Table 1, with the arterial line filter purge turned off. Table 2 shows the same experimental conditions but with the arterial line filter purge in the clinically recommended open position. Effective forward flow is negatively impacted with increasing levels of VAVD, with and without the arterial line filter purge in the open position, as shown in Tables 1 and 2. The use of the highest experimental VAVD setting caused a significant (*P*<0.0001) reduction of the arterial flow from 1490±0.00 mL/min to 590±0.00 mL/min, from 2020±0.01 mL/min to 1220±0.00 mL/min, and from 2490±0.0 mL/min to 1830±0.01 mL/min, as shown in Figure 2, graphics I and II. A significant (*P*<0.0001) and progressive decrease in effective forward flow was also observed when the purge line was open, as shown in Figure 3, graphic III. Shunt flow with the arterial filter purge line open progressively increased (*P*=0.0003) as the vacuum applied to the venous reservoir also increased. Purge line shunt flow was proportionally higher with lower pump flows and increasing levels of VAVD, ranging from 80% of overall flow with the arterial flow set at 1500 mL/min with -80 mmHg of VAVD to 34% of overall flow with the arterial flow set at 2500 mL/min with -80 mmHg, as shown in Figure 3, graphic IV (*P*=0.01).

## DISCUSSION

VAVD is a common technique used in many cardiac surgery centers worldwide^[11-13]^. Its use seeks to significantly improve venous drainage during CPB surgeries, especially when smaller inner diameter cannulas are used or with minimally invasive surgery^[14,15]^. Applying vacuum to a sealed venous reservoir improves venous drainage, but it also impacts CP output when one is used in the arterial position. Increased VAVD levels require the clinician to increase the CP rotations per minute (RPM) to compensate^[16]^. We found a significant decrease in effective forward flow from 1490 to 590 mL/min, from 2020 to 1220 mL/min, and from 2490 to 1830 mL/min with the application of -80 mmHg of VAVD in our experimental circuit. With the arterial filter purge line open or with any shunt line, such as a passive hemoconcentrator, increasing VAVD levels result in increased shunt flow, which steals effective forward flow for the patient^[16]^. Therefore, using a flow probe is essential for patient safety, particularly with external arterial line filters and a CP in the arterial position^[16]^.

The main finding in this experiment was that effective forward flow is compromised both with the use of a centrifugal arterial pump head and an open external arterial line filter purge and that these effects are magnified with increasing levels of VAVD. This is of concern for clinical practice in Brazil, as many centers use a centrifugal arterial pump head without monitoring effective forward flow with an independent flow probe as we did in this experiment.

Brazil has a long history of creating innovative products for cardiovascular surgery, including domestically produced heart-lung machines (HLMs), oxygenators, and other perfusion products. While Brazil maintains an environment of innovation, there are areas of clinical practice where outdated technologies are still clinically employed^[17]^. Some perfusionists and surgeons consider Brazilian non-servo-controlled HLMs unsafe for pediatric perfusion, especially when low flow is needed. Some also prefer using an imported CP as the main arterial pump. For them, using CP as the main pump, despite any scientific data, is related to safer and better outcomes.

In the literature, controversy persists regarding the advantages and preferences of using RP *vs*. CP for generating arterial flow^[18]^. However, the lack of clarity of clinical benefits of the more expensive CP is exemplified in the significant disparity in the choice of main arterial pump in North America (RP = 51%, CP = 49%), Australia-New Zealand (RP = 70%, CP = 30%), and Europe (RP = 90%, CP = 10%)^[5,19]^. A recent survey in Brazil^[9]^ showed that CPs are used as the main arterial pump in cardiac surgeries, including pediatric and neonatal patients, in 60% of cardiac centers. There have been conflicting results reporting the effects of the two pump types on transfusions, platelet counts, plasma-free hemoglobin, postoperative mediastinal bleeding, and clinical outcomes^[5-7]^. Studies comparing CP and RP generally have small sample size with limited statistical power to detect differences in outcome measures. A meta-analysis of randomized controlled trials has been performed to combine the clinical outcome data and synthesize the evidence comparing RP and CP as the main arterial pump in adult cardiac surgery^[8]^. No differences in postoperative blood loss, hospital length of stay, intensive care unit length of stay, mortality, or device mishap-related morbidity or hematologic variables were found^[8]^. These findings suggest that RPs and CPs can both be used with comparable outcomes.

One major concern is that CP is afterload-dependent. An increase in downstream resistance decreases forward flow delivered to the patient if no adjustment is made to the set RPM^[20]^. Furthermore, a CP is preload-dependent and any modification in the pressure at the CP inflow will impact pump output, as shown in our *in vitro* experiment. Therefore, effective forward flow with the use of a CP for the arterial pump is impacted by both inflow and outflow conditions.

Literature has shown that augmented venous return techniques may help to introduce GME into the patient undergoing CPB^[15,16,21]^. Although many other potential causes for gaseous emboli during CPB have been identified (venous reservoir with low level of blood, cavitation, etc.), the creation of a negative pressure in the venous line facilitates entrainment of air around the venous cannula, possibly increasing GME^[21]^. LaPietra et al.^[22]^ compared the potential for GME formation and transmission with the use of augmented venous return systems and the GME handling capabilities of various clinical augmented venous return CPB circuits. Significant increases were associated with increasing negative vacuum pressure (*P*<0.001) and use of an arterial RP (*P*=0.01). They concluded that when assisted venous drainage techniques are used, a CP as the main arterial pump enhances the removal of significant GME that may occur even in the presence of a standard arterial line filter^[22]^. Despite this potential advantage of using CP and VAVD, we did not assess GME in our experiment.

The use of combined methods, CP and VAVD, is safe, but it requires caution and an adequate arterial flow to help ensure effective forward flow and end-organ perfusion.

It is worth noting that the purge line flow is limited by the size of the purge line tubing. This explains the findings that similar purge line flows obtained at increasing CP flows. Consequently, purge line flow may have a greater impact when lower flows are required.

This study has the limitations of an in vitro experiment. In vivo studies may be needed to evaluate the impacts of these findings on clinical outcomes.

## CONCLUSION

The use of VAVD reduces the effective arterial flow when a CP is used in the arterial position and this flow reduction is amplified when an external arterial line filter is in use. All perfusion systems should utilize an independent flow probe on the arterial limb, and this is especially essential with the use of a centrifugal arterial pump head, with and without an external arterial line filter. The use of a flow probe, as shown in our simulated pediatric bypass circuit, will help ensure effective forward flow and adequate systemic perfusion during CPB.

**Table:** 

Author's roles & responsibilities	
DPG	Substantial contributions to the conception or design of the work; or the acquisition, analysis, or interpretation of data for the work; drafting the work or revising it critically for important intellectual content; final approval of the version to be published;
LFC	Substantial contributions to the conception or design of the work; or the acquisition, analysis, or interpretation of data for the work; drafting the work or revising it critically for important intellectual content; final approval of the version to be published;
GM	Drafting the work or revising it critically for important intellectual content; final approval of the version to be published
LPC	Substantial contributions to the conception or design of the work; or the acquisition, analysis, or interpretation of data for the work; drafting the work or revising it critically for important intellectual content; final approval of the version to be published
VCP	Substantial contributions to the conception or design of the work; or the acquisition, analysis, or interpretation of data for the work
AVCXC	Substantial contributions to the conception or design of the work; or the acquisition, analysis, or interpretation of data for the work
MHCM	Substantial contributions to the conception or design of the work; or the acquisition, analysis, or interpretation of data for the work
PSC	Substantial contributions to the conception or design of the work; or the acquisition, analysis, or interpretation of data for the work
MBJ	Final approval of the version to be published
IC	Substantial contributions to the conception or design of the work; or the acquisition, analysis, or interpretation of data for the work; drafting the work or revising it critically for important intellectual content; final approval of the version to be published;
FBJ	Final approval of the version to be published
